# Supplementary material for: The causal role of circulating inflammatory markers in osteoporosis: a bidirectional Mendelian randomized study
Source: Front Immunol. 2024 Jul 18;15:1412298. doi: 10.3389/fimmu.2024.1412298 (PMC11291241; doi:10.3389/fimmu.2024.1412298)
Supplement: Supplementary file 1 [file DataSheet_1.zip › supplementary material/CIM-OSTEOPOROSIS /GCST90274812.csv_leave_one_out.pdf]

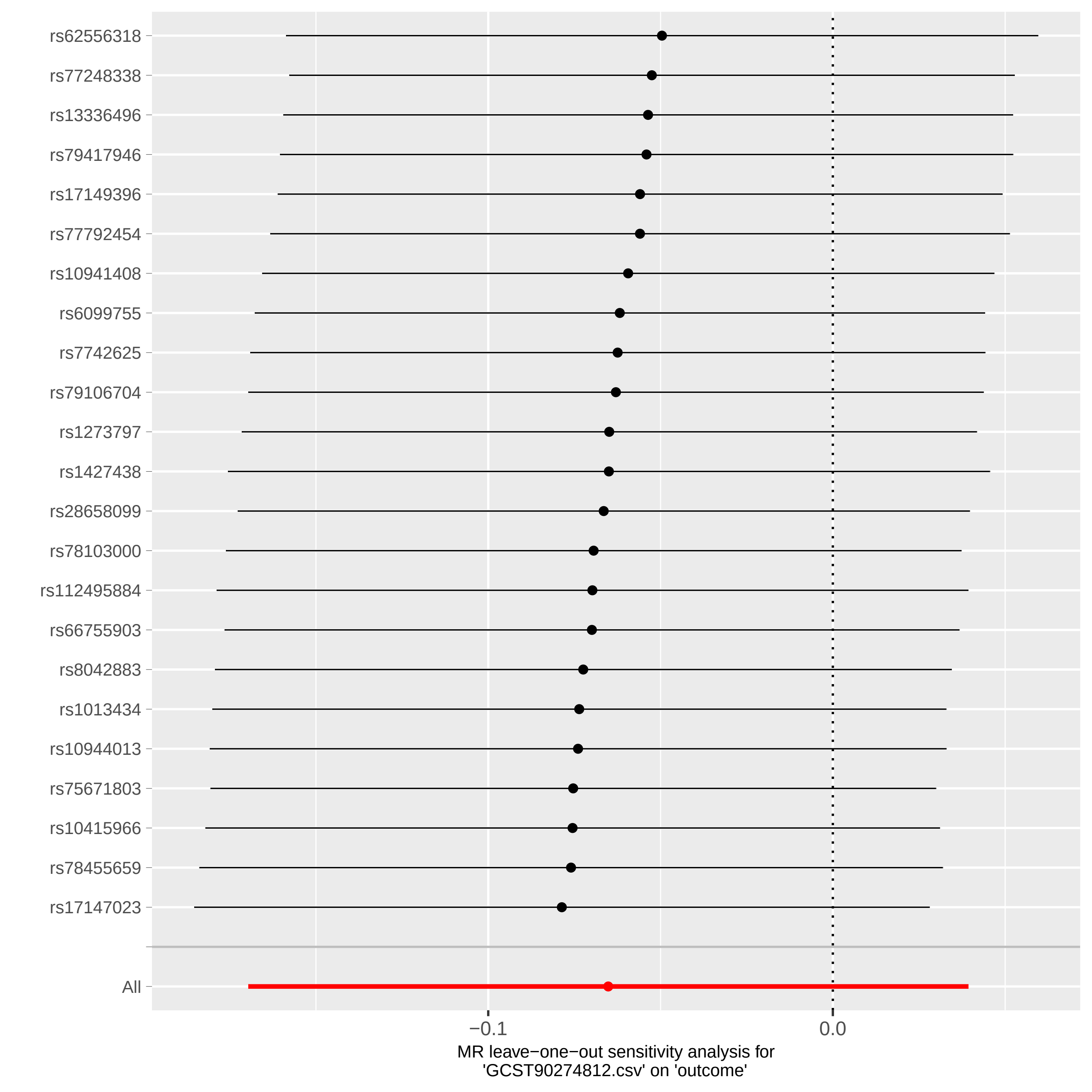

rs62556318
rs77248338
rs13336496
rs79417946
rs17149396
rs77792454
rs10941408
rs6099755
rs7742625
rs79106704
rs1273797
rs1427438
rs28658099
rs78103000
rs112495884
rs66755903
rs8042883
rs1013434
rs10944013
rs75671803
rs10415966
rs78455659
rs17147023
All
−0.1
0.0
MR leave−one−out sensitivity analysis for
'GCST90274812.csv' on 'outcome'
